# Impact of parathyroidectomy among nondiabetic hemodialysis patients with severe hyperparathyroidism

**DOI:** 10.1080/0886022X.2022.2098768

**Published:** 2022-07-26

**Authors:** Qing-xiu Huang, Jie Pang, Chuan-ke Shi, Xiao-wen Huang, Xiao-fang Chen, Yan-feng Luo, Hai-wen An, Jian-lin Jian, Linna Liu, Yan-lin Li

**Affiliations:** a Department of Nephrology, Zhongshan Hospital of Traditional Chinese Medicine, Guangzhou University of Chinese Medicine, Zhongshan, China; b Department of Surgery, Zhongshan Hospital of Traditional Chinese Medicine, Guangzhou University of Chinese Medicine, Zhongshan, China; c Department of Ultrasonography, Zhongshan Hospital of Traditional Chinese Medicine, Guangzhou University of Chinese Medicine, Zhongshan, China

**Keywords:** Parathyroidectomy, hyperparathyroidism, hemodialysis, outcome

## Abstract

**Background:**

Parathyroidectomy (PTX) is a treatment for hyperparathyroidism (HPT) and has uncertain risks and benefits. The aim of this study was to evaluate the effect of PTX versus nonoperative treatment among nondiabetic hemodialysis patients.

**Methods:**

A retrospective matched cohort study was performed. Each PTX patient was matched with one patient who had severe HPT but rejected PTX. The patients were matched by sex, birth date, date of first dialysis, nondiabetic status, and left ventricular ejection fraction. The serum markers, survival, main adverse cardiovascular and cerebrovascular event (MACCE) rates, and hospitalization were compared between the PTX patients and matched non-PTX patients.

**Results:**

There were 1143 patients at our center in the Chinese National Renal Data System (CNRDS) between 2010 and 2020. Of these, 75 PTX patients were matched with 75 non-PTX patients. Rapid decreases in the mean intact parathyroid hormone, calcium and phosphorus concentrations, and a gradual increase in hemoglobin concentration were observed in the PTX group. The mortality was 2.9 per 100 patient-years in the PTX group and 10.9 per 100 patient-years in the non-PTX group (*p* < 0.001). Compared with non-PTX patients, PTX patients had an adjusted HR for death of 0.236 (95% CI 0.108–0.518). The cumulative MACCE rates were 6.7 per 100 patient-years in the PTX group and 15.2 per 100 patient-years in the non-PTX group (*p* < 0.001). The adjusted HR of the occurrence of first MACCE for PTX patients compared with non-PTX patients was 0.524 (95% CI 0.279-0.982). The cumulative hospitalization rates were 50.3 per 100 patient-years in the PTX group and 66.5 per 100 patient-years in the matched non-PTX group (*p* < 0.001).

**Conclusions:**

Compared with non-PTX patients, PTX was associated with an improvement in the biochemical measures and patient-level outcomes in nondiabetic hemodialysis patients with severe HPT.

## 1 Introduction

The mineral and bone disorder of chronic kidney disease (CKD–MBD), a common complication of end-stage renal disease (ESRD), has been an area of intense interest and controversy over the past few years. A large number of observational studies have demonstrated that severe hyperparathyroidism (HPT) is associated with anaemia [1], mineral and bone disorder [2], vascular calcifications and calciphylaxis [3], cardiovascular diseases [4,5] and mortality [4,6]. Thus, for dialysis patients, the Kidney Disease Improving Global Outcomes (KDIGO) guidelines recommend a parathyroid hormone (PTH) level target of two to nine times the upper limit of normal [7]. The available therapies for secondary HPT include cinacalcet, calcitriol, and parathyroidectomy (PTX). However, all of the treatments remain controversial, and the best option remains unknown [7].

The KDIGO guidelines currently state that PTX remains a valid treatment option, especially when medical treatment fails [7]. However, PTX is an invasive procedure with uncertain risks and benefits. Multiple previous studies have shown that PTX results in a marked reduction in the levels of serum PTH, calcium, and phosphorus [8–10]. However, there are no high-quality studies that demonstrate a clear beneficial effect of PTX on patient-level outcomes. Some [11–18] but not all [19–21] previous studies have shown that PTX is associated with an improvement in survival. These studies were contradictory and were limited in some factors, such as the race of the population, the short-term follow-up [17], and the possibility for patient selection bias [12–14]. Some studies had no control group [21] or used general dialysis patients instead of severe HPT patients as a control group [12]. A few studies have focused only on nondiabetic patients [18].

## 2 Materials and methods

### 2.1. Study cohort

The study was approved by the review board of Guangzhou University of Chinese Medicine (approval No.2022ZSZY-LLK-264) and complied with the Declaration of Helsinki. The requirement for informed consent was waived because the study was retrospective. This retrospective observational matched cohort study included all maintenance hemodialysis patients in the Chinese National Renal Data System (CNRDS) who were registered by our center between 10 May 2010 and 31 December 2020. Patients were excluded for the following reasons: missing follow-up data, without severe HTP (PTH < 600 pg/ml), and underwent PTX before the study. Full-time staff was responsible for the system information registration and all of the hemodialysis patients’ follow-up in our center. Hence, the data of the cohort were relatively complete and reliable.

### 2.2. PTX group

Data on both total PTX (with forearm transplantation) and subtotal PTX were included in the study. The PTX data were retrieved from our inpatient system and then compared with the data from the CNRDS. The indications for PTX in our center included persistently intact PTH levels higher than 600 pg/ml and refractoriness to medical treatment, uncontrolled hypercalcaemia, hyperphosphatemia, or clinical symptoms of severe HPT[7]. The common contraindication of PTX in our center is a left ventricular ejection fraction (LVEF) <30%. All surgeries were performed by the same experienced surgeon. We had a team for the patients’ perioperative and postoperative follow-up care.

To our surprise, only one of the 81 hemodialysis patients undergoing PTX in our center had diabetes mellitus. Thus, we decided to perform a study focusing on only nondiabetic patients.

### 2.3. Matching the non-PTX group

The patients who underwent PTX therapy in the cohort were matched with one patient who had severe HPT (at least one intact PTH measurement ≥600 pg/ml), but who had rejected PTX. The patients were matched using the following criteria: sex, birth date (in five-year categories), date of first dialysis (in five-year categories), without diabetes mellitus, and with a left ventricular ejection fraction (LVEF) ≥30%.

To calculate the survival time, the matched non-PTX patients were assigned the calendar date of surgery of the respective matched PTX patients, and this date was hereafter referred to as PTX(d). The matched non-PTX patients were required to be alive on this particular date.

### 2.4. Outcomes and exposures

The follow-up started on the date of PTX(d) and ended on the date of death or 31 December 2021, whichever came first. The biochemical changes associated with PTX were measured. The primary outcome was all-cause mortality. The secondary outcomes were main adverse cardiovascular and cerebrovascular events (MACCE), fracture, and hospitalization. In this study, the MACCEs included cerebral hemorrhage, stoke, heart failure, myocardial infarction, unstable angina, peripheral vascular events, and sudden death [22]. The outcome data were retrospectively retrieved from the CNRDS and inpatient systems.

### 2.5. Statistical analysis

All statistical analyses were performed using IBM SPSS (version 22.0) and GraphPad Prism (version 9.0). The data with a normal distribution are expressed as the mean ± standard deviation. The parameters without a normal distribution are expressed as the median with range. Numbers (percentages) were calculated for categorical variables. We compared the baseline characteristics according to the categories by using *t* tests, χ^2^ tests, and Kruskal–Wallis tests.

The χ^2^ test was used to compare the exposure-adjusted rate of the patient-level events between the PTX and matched non-PTX patients. The Kaplan–Meier method was used to evaluate the effect of PTX versus nonoperative treatment on death and the occurrence of first MACCE, using the time period from time PTX(d) to censoring as the timescale. Univariate and multivariate Cox proportional hazards regression models were used to compare the hazard ratios (HRs) with 95% confidence intervals (CIs) for death or the occurrence of first MACCE between the PTX and matched non-PTX patients, using the time from PTX(d) to censoring as the timescale. The results with a *p* value <0.05 were considered statistically significant.

## 3 Results

### 3.1. Patients and data

**Figure 1.**
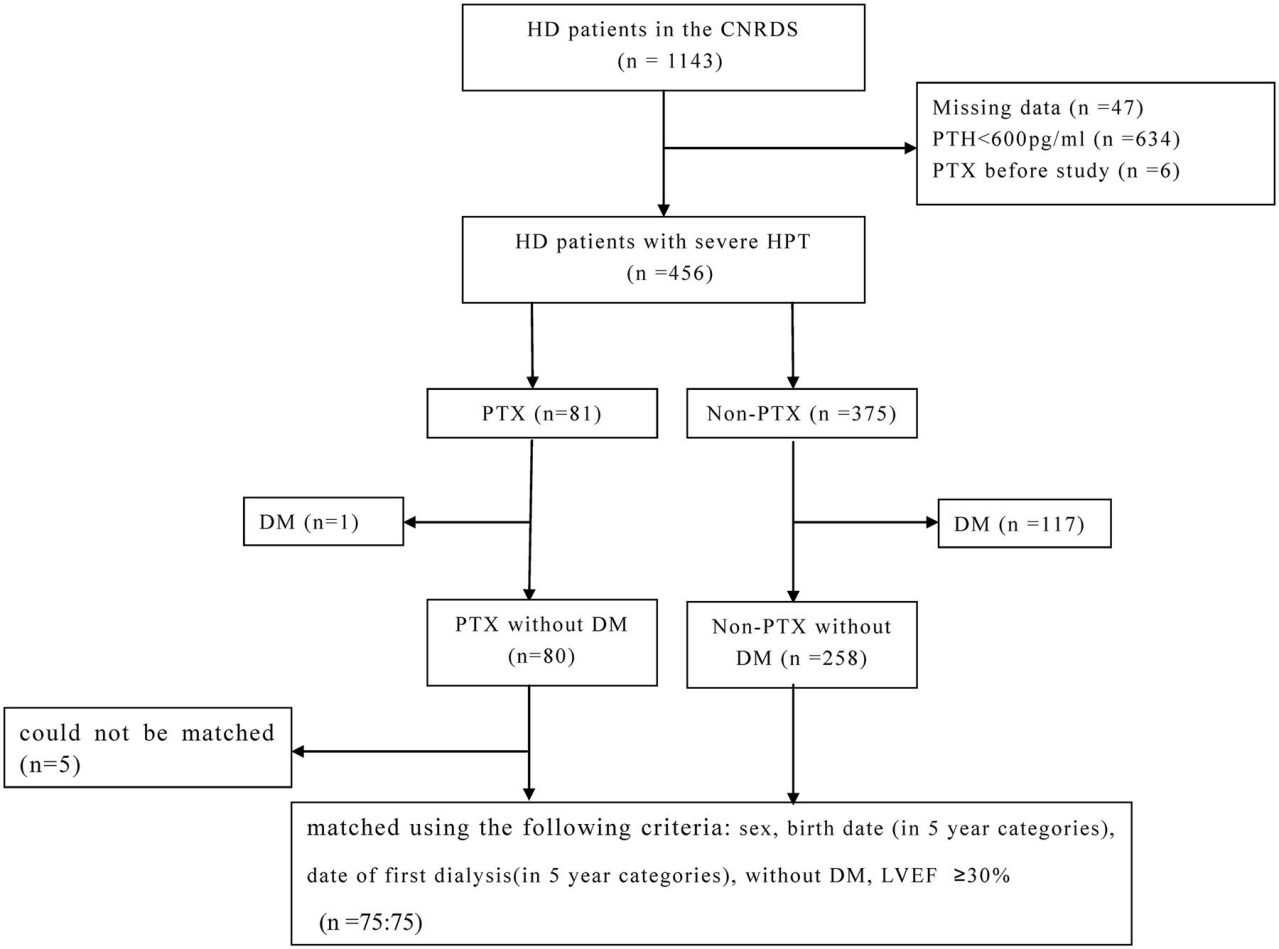
Study profile. CNRDS, Chinese National Renal Data System; PTH, parathyroid hormone; PTX, parathyroidectomy; HPT, hyperparathyroidism; HD, hemodialysis; PD, peritoneal dialysis; DM, diabetes mellitus; LVEF, left ventricular ejection fraction.

The study cohort profile is shown in Figure 1. There were 1143 patients in the CNRDS registered by our center during the study period. Of these, a total of 687 patients were excluded for the following reasons: missing data (*n* = 47), not having severe HPT (PTH < 600 pg/ml) (*n* = 634), and undergoing PTX before the study (*n* = 6). Among the 456 patients with severe HPT, 81 had undergone PTX and 375 did not undergo PTX.

Unexpectedly, only one of 81 PTX patients had diabetes mellitus and was excluded. Similarly, 117 diabetic patients were excluded in the non-PTX group. Five PTX patients could not be matched according to our matching criteria and were excluded. The 75 patients with PTX were matched with 75 non-PTX patients.

### 3.2. Patient characteristics at baseline

The baseline characteristics of the PTX patients and matched non-PTX patients are shown in Table 1. There were no significant differences in sex, age, dialysis vintage, causes of end-stage renal disease (ESRD), or Charlson comorbidity (CCI) scores at the time of the PTX (d) between the two groups. The mean baseline intact PTH level of the PTX group was higher than that of non-PTX(*p* = 0.043). However, the baseline values for calcium, phosphorus, albumin, alkaline phosphatase (ALP), beta 2 microglobulin (β2MG), single-pool Kt/V, urea reduction ratio (URR), interdialysis weight gain (IDWG), and body mass index (BMI) were comparable between the two groups.

**Table 1.** Baseline of matched PTX and non-PTX patients at PTX(d).^#^

Characteristics	PTX	Non-PTX	*p* value
Female/male	30/45	30/45	N.S.
Age at PTX (d)^#^ (years)	48.0 ± 11.1	47.9 ± 11.1	0.973
Age at start of hemodialysis (years)	40.9 ± 10.7	41.6 ± 11.3	0.712
Dialysis vintage (months)	72.8 (54.1, 109.1)	69.3 (48.3, 101.4)	0.271
Causes of ESRD (%)			0.331
Chronic glomerulonephritis	65 (86.7%)	58 (77.3%)	
Lupus nephritis	3(4%)	5(6.7%)	
Others or unknow	7(9.3%)	12(16.0%)	
CCI score (%)			0.645
1	48 (64.0%)	53 (70.7%)	
2	21 (28.0%)	18 (24.0%)	
3	6 (8.0%)	4 (5.3%)	
intact PTH (pg/ml)	1673 ± 375	699 ± 516	**0.043**
Previous fracture	10 (13.3%)	6 (8.0%)	0.428
Calcium (mmol/L)	2.45 ± 0.23	2.18 ± 0.24	0.740
Phosphorus (mmol/L)	2.39 ± 0.52	2.35 ± 0.55	0.749
Albumin (g/L)	39.2 ± 2.76	38.0 ± 3.2	0.381
Serum creatinine (umol/L)	1141 ± 272	1099 ± 238	0.610
BUN (mmol/L)	26.7 ± 7.8	26.2 ± 6.3	0.059
ALP (U/L)	80.6 ± 42.1	101.2 ± 40.4	0.562
LDL-c (mmol/L)	2.43 ± 0.74	2.26 ± 0.80	0.687
β2MG	23.2 ± 7.5	24.5 ± 8.1	0.160
SpKt/V	1.44 ± 0.23	1.50 ± 0.24	0.802
URR	0.70 ± 0.06	0.71 ± 0.06	0.873
IDWG	0.044 ± 0.019	0.047 ± 0.016	0.356
BMI (Kg/m^2^)	23.0 ± 3.3	21.9 ± 3.0	0.414

PTX, parathyroidectomy; N.S, no significance; ESRD, end-stage renal disease; CCI score, Charlson comorbidity score; PTH, parathyroid hormone; BUN, blood urea nitrogen; ALP, alkaline phosphatase; LDL-c, low density lipoprotein cholesterol; β2MG, beta 2 microglobulin; SpKt/V, single-pool Kt/V; URR, urea reduction ratio; IDWG, interdialysis weight gain; BMI, body mass index.

^#^The date of PTX or corresponding time for non-PTX patients.

Bold values indicates significant statistical differences.

### 3.3. Changes in the biochemical measures

**Figure 2.**
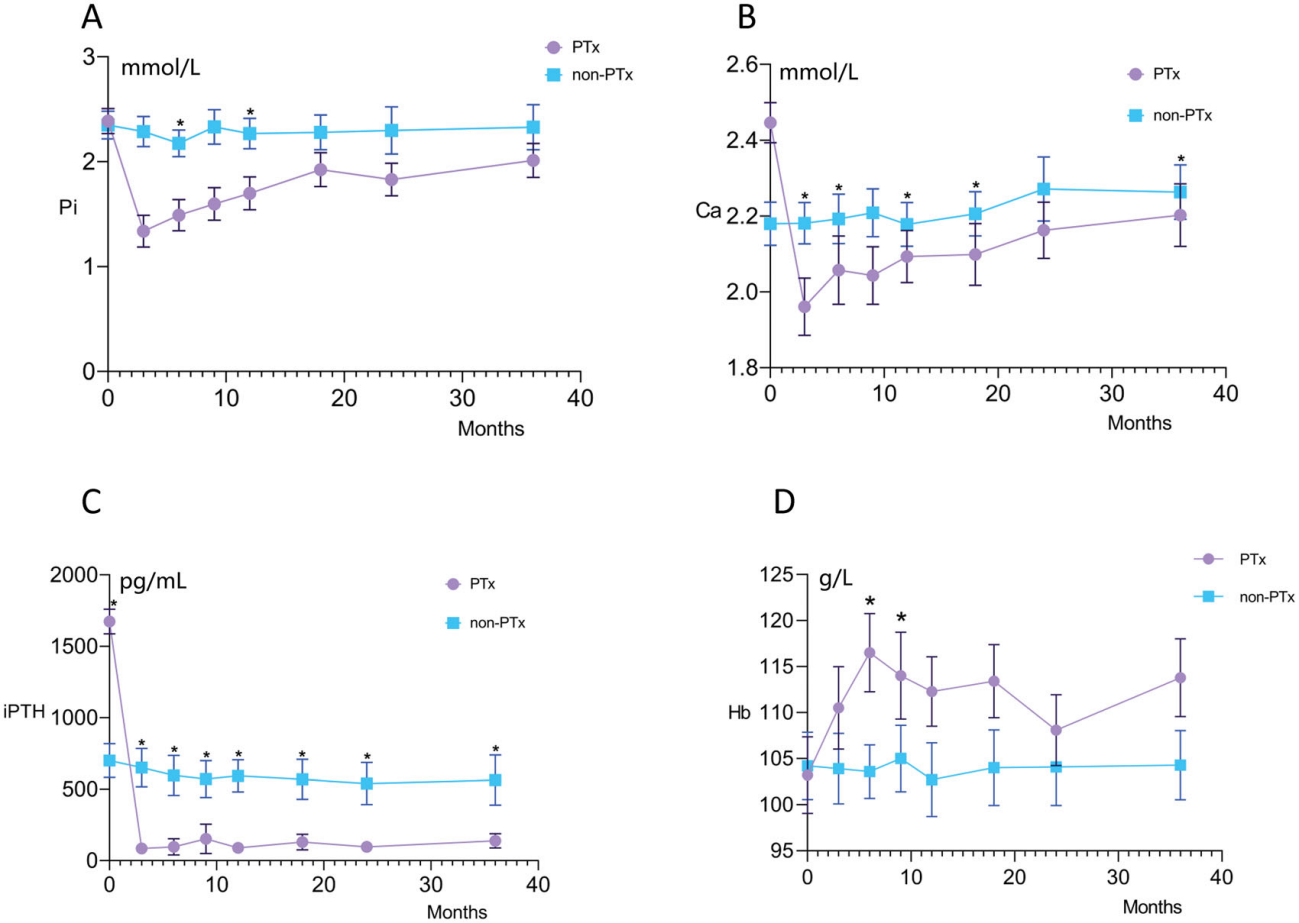
The mean serum levels of intact PTH, calcium, phosphorus, and hemoglobin in the PTX and non-PTX groups over time. PTX, parathyroidectomy; Pi, phosphorus; Ca, calcium; iPTH, intact parathyroid hormone; Hb, hemoglobin. * *p* value <0.05

The mean serum levels of intact PTH, calcium, phosphorus, and hemoglobin in the two groups over time are shown in Figure 2. Rapid decreases in the mean intact PTH, calcium, and phosphorus concentrations were observed in the PTX group. The mean intact PTH, calcium, and phosphorus concentrations of the PTX patients remained lower than those of the non-PTX patients over the three-year follow-up period. In addition, a gradual increase in the mean hemoglobin concentration was observed in the PTX group compared with the non-PTX group (Figure 2(d)).

### 3.4. Patient-Level outcomes

#### 3.4.1. Mortality

The median follow-up period was 52.5 months for the PTX patients and 33.2 months for the non-PTX patients. A total of 10 (13.3%) PTX patients died. Five died from cerebrovascular events, two died from infection, and three died from other etiologies or had an unknown cause of death. A total of 28 (37.3%) non-PTX patients died. Sixteen died from cerebrovascular events, five died from infection, and seven died from other etiologies or had an unknown cause of death. As shown in Table 2, the exposure-adjusted mortality was 2.9 per 100 patient-years in the PTX group and 10.9 per 100 patient-years in the matched non-PTX group (*p* < 0.001).

**Table 2.** Patient-level outcomes of PTX patients and matched non-PTX patients.

Events	PTX (*n* = 75, 342 patient-years)			non-PTX (*n* = 75, 257 patient-years)		*p* *
	No. of Events	Exposure-Adjusted Rate(per 100 patient-year)^#^		No. of Events	Exposure-Adjusted Rate(per 100 patient-year)^#^	
Death	10	2.9		28	10.9	**<0.001**
MACCE	23	6.7		39	15.2	**<0.001**
Hospitalization	172	50.3		171	66.5	**<0.001**
Fracture	4	1.2		3	1.2	0.998

MACCE, main adverse cardiovascular and cerebrovascular events; PTX, parathyroidectomy.

*p values were calculated for the exposure-adjusted incidence rate.

^#^The exposure-adjusted rate was calculated as 100 times the total number of events divided by the total number of patient-years of exposure.

Bold values indicates significant statistical differences.

Both univariate and multivariate Cox survival analyses indicated that PTX was associated with better survival (Table 3). The crude HR for death was 0.233 (95% CI 0.109–0.496) for the PTX patients compared with the non-PTX patients. In addition, older age at PTX(d) and higher CCI scores were associated with higher mortality in the crude analyses. After adjustment for sex, age at PTX (d), dialysis vintage, cause of ESRD and CCI score at PTX (d), the adjusted HR for death after PTX was 0.236 (95% CI 0.108–0.518).

**Table 3.** Cox regression of the effect of PTX on death and the occurrence of first MACCE.

Variables	Death		MACCE	
	crude HR	adjusted HR	crude HR	adjusted HR
PTX	0.233 (0.109, 0.496)	**0.236 (0.108, 0.518)**	0.500 (0.267, 0.936)	**0.524 (0.279, 0.982)**
Male	1.927 (0.935, 3.972)	**3.145 (1.324, 7.470)**	1.301 (0.688, 2.462)	1.499 (0.756, 2.969)
Age at PTX (d),(per↑1 year)	1.047 (1.015, 1.080)	**1.051 (1.018, 1.084)**	1.037 (1.007, 1.067)	**1.037 (1.007, 1.068)**
Dialysis vintage,(per↑1 year)	0.999 (0.993, 1.006)	1.003 (0.996, 1.010)	1.000 (0.994, 1.006)	
Cause of ESRD				
chronic glomerulonephritis	ref.		ref.	
lupus nephritis	1.947 (0.673, 5.637)	2.975 (0.778, 11.375)	1.478 (0.446, 4.899)	1.750 (0.458, 6.688)
Others or unknown	1.355 (0.556, 3.301)	1.430 (0.571, 3.581)	1.391 (0.611, 3.169)	1.319 (0.576, 3.018)
CCI score at d,(per↑1)	1.728 (1.113, 2.684)	**1.971 (1.710, 3.319)**	1.252 (0.783, 2.004)	1.309 (0.778, 2.204)

MACCE, main adverse cardiovascular and cerebrovascular events; HR, hazard ratio; PTX, parathyroidectomy; ESRD, end-stage renal disease; CCI score, Charlson comorbidity score.

Bold values indicates significant statistical differences.

**Figure 3.**
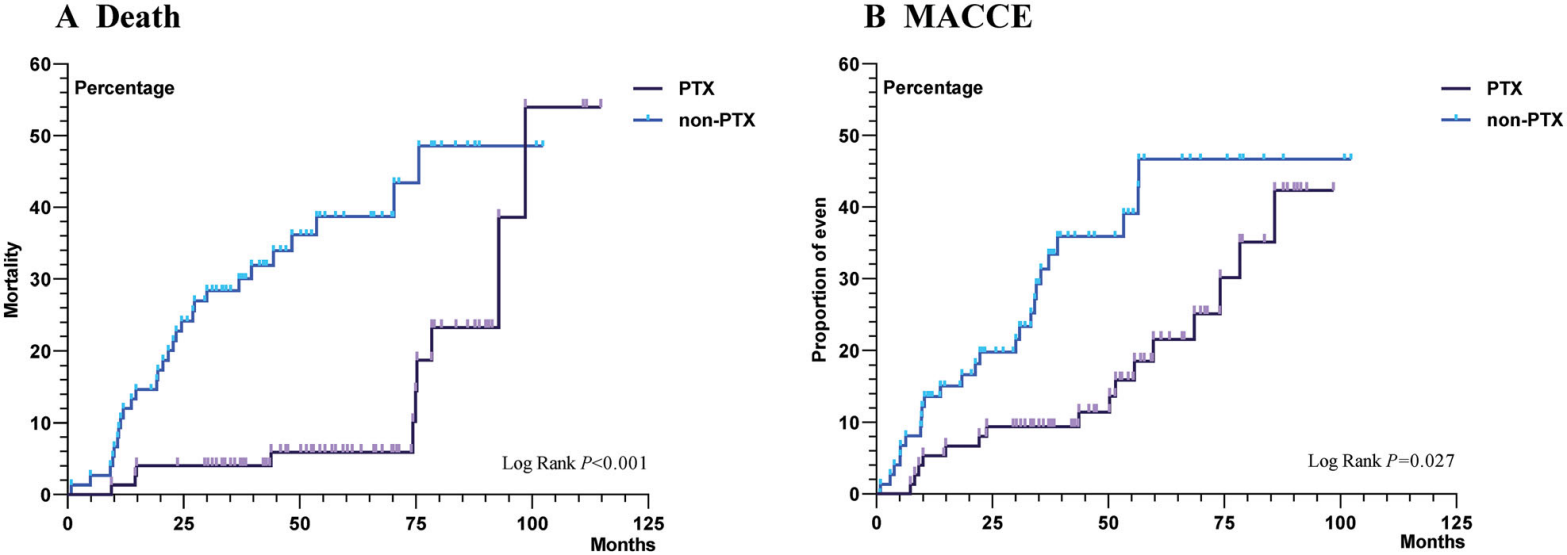
Kaplan–Meier survival and MACCE estimates for the PTX and non-PTX patients. MACCE, main adverse cardiovascular and cerebrovascular events; PTX, parathyroidectomy.

The Kaplan–Meier survival curve also showed better survival for the PTX patients than the matched non-PTX patients (log-rank *p* < 0.001) (Figure 3(a)). The median survival time after PTX(d) was 98.5 months for the PTX patients and 75.6 months for the non-PTX patients. None of the patients who underwent PTX during the study died within the first 30 days.

#### 3.4.2. Main adverse cardiovascular and cerebrovascular events (MACCEs)

As shown in Table 2, the cumulative MACCE rates were 6.7 per 100 patient-years in the PTX group and 15.2 per 100 patient-years in the matched non-PTX group (*p* < 0.001). The amount of MACCEs in the PTX group was as follows: eight episodes of heart failure, five episodes of stroke, three cases of encephalorrhagia, two myocardial infarctions, and five other events. In the matched non-PTX group, the amount of MACCEs was as follows: 12 episodes of stroke, 8 episodes of heart failure, 6 myocardial infarctions, 5 cases of encephalorrhagia, and 8 other events.

The crude HR and adjusted HR of the occurrence of first MACCE in the PTX patients compared with the non-PTX patients were 0.500 (95% CI 0.267–0.936) and 0.524 (95% CI 0.279–0.982), respectively (Table 3).

The Kaplan–Meier curve also showed that PTX was associated with a lower risk of the occurrence of a first MACCE. (log-rank *p* = 0.027) (Figure 3(b)).

#### 3.4.3. Hospitalization

The cumulative hospitalization rates were 50.3 (95% CI, 38.2–62.0) per 100 patient-years in the PTX group and 66.5 (95% CI, 50.2–83.6) per 100 patient-years in the matched non-PTX group, and this was a nominally significant result (*p* < 0.001) (Table 2).

#### 3.4.4. Fracture

Fractures occurred in four patients in the PTX group and three patients in the non-PTX group, and both of these numbers corresponded to exposure-adjusted rates of 1.2 events per 100 patient-years (Table 2). There were no significant effects of PTX on the risk of fracture (*p* = 0.998).

## 4 Discussion

In this retrospective observational matched cohort study, PTX was demonstrated to be associated with an improvement in biochemical measures, decreased all-cause mortality, decreased MACCE rates, and decreased cumulative hospitalization rates in nondiabetic hemodialysis patients with severe HPT. However, PTX did not demonstrate a protective effect against fracture in this study. These data provide new evidence regarding positive patient-level outcomes related to PTX.

At present, there are no randomized trials that have been performed to compare PTX with medical treatment alone. The KDIGO guideline working groups recommend PTX when PTH-lowering medicines fail. In other words, medicine is preferred to surgery most of the time. Thus, the PTX rates in dialysis patients have declined markedly over the past few decades and have ranged from a high of 12.5 per 1000 patient-years in 1992 to a low of 5.5 per 1000 patient-years in 2005 in the US [23,24]. However, due to economic reasons, PTX remains a relatively common treatment option in our center. Our research suggested that PTX is an effective treatment because of the improvement in the biochemical parameters as well as the patient-level outcomes in this study.

The effects of PTX on long-term survival are still subject to controversy. The study by Kestenbaum et al. showed that the long-term mortality was lower in patients undergoing PTX compared with a matched cohort during a median 2.9-year follow-up time [14]. Another large sample size study by Komaba et al. showed that patients undergoing PTX had a lower risk for mortality than the matched controls during one year of follow-up [17]. However, Trombetti et al. found a lower mortality rate after PTX, but this lower mortality rate disappeared after adjusting for comorbidities [19]. Moreover, Fotheringham et al. found that the patients within the lowest quartile of iPTH recovery in the five years following PTX were associated with poorer patient survival [25]. These studies were contradictory and were also limited in some factors, such as the race and region of the patients, the short-term follow-up, and the potential of selection bias in the studies. We performed a long-term retrospective nested index-referent study to verify that PTX was associated with a reduced risk of morbidity in nondiabetic hemodialysis patients in our single center.

The effects of PTX on 30-day postoperative survival are still subject to controversy. In this study, none of the patients died within the first 30 days after surgery. Some previous studies showed that the 30-day postoperative mortality rate following PTX was as high as 2.0–3.1% [14,21]. However, some studies have shown that PTX is safe and does not result in postoperative mortality [26,27]. The patient selection, the regional differences in referral for PTX, the proficiency of the surgeons, and variability arising from the small sample sizes likely account for this variation.

The effects of PTX on patient-level outcomes in this study were contrary to the study by Ishani et al. A nationwide cohort study in the USA by Ishani et al. showed that the 30-day postoperative mortality rate following PTX was as high as 2.0%, hospitalizations were higher in the year after PTX, and the cause-specific hospitalizations were higher for patients with acute myocardial infarction and dysrhythmia. This was a pre-post design study that was different from our nested index-referent study. Our study showed that the cumulative hospitalization rate and number of cardio-cerebrovascular events were lower in patients undergoing PTX than in the matched group. The surgical experience and the team responsible for postoperative follow-up were high quality in our single center, which might account for the different results of this single center study compared with the nationwide cohort study. In addition, the Medicare policy, regional differences, and patient selection made a difference in the results in this study.

Consistent with many previous studies [28–31], our study shows that PTX was associated with not only calcium, phosphorus, and iPTH levels but also hemoglobin levels. In addition, some previous studies demonstrated a decrease in erythropoietin(EPO) requirements after PTX [32–34]. Hyperparathyroidism may cause fibrocystic osteitis and interfere with the generation and survival of red blood cells [35]. The physiological responses to PTX are likely multifactorial and potentially increase the bioavailability of EPO [32].

Only one of the 81 hemodialysis patients who underwent PTX in our center had diabetes mellitus. One of the reasons might be patient selection by the surgeon since diabetic patients may have more comorbidities [36–38]. Another reason might be that hemodialysis patients with diabetes have shorter survival times than nondiabetic hemodialysis patients [39–41]. The median dialysis vintage of PTX patients was 72.8 months in our study. Hemodialysis patients with diabetes in our center had an approximately five-year median survival time in our preliminary observations.

There are several limitations of this study. The main limitation is that it is not a randomized study but a retrospective cohort study. Despite matching and adjustment for several confounding factors, residual confounding cannot be excluded. Second, we did not investigate the drug treatment data in the non-PTX patients, which could have some impact on the results. In addition, patients undergoing PTX might receive more medical care than nonsurgical patients. Third, the two groups were not well matched as the PTX patients had higher iPTH levels than did the non-PTX patients. Fourth, it is a single-center study, and the surgical experience and follow-up after PTX may also make a difference in the results.

## 5 Conclusion

In conclusion, our study suggested that PTX was associated with an improvement in biochemical measures and patient-level outcomes in nondiabetic hemodialysis patients with severe HPT. PTX may be considered a therapy of priority in centers with high-quality surgical experience and follow-up team.

## Data Availability

The datasets used and/or analyzed during the current study are available from the first author on reasonable request.

## Abbreviations

ALP: Alkaline phosphatase
β2MG: Beta 2 microglobulin
BMI: Body mass index
CCI: Charlson comorbidity
CIs: Confidence intervals
CKD–MBD: Mineral and bone disorder of chronic kidney disease
CNRDS: Chinese National Renal Data System
ESRD: End-stage renal disease
HPT: Hyperparathyroidism
IDWG: Interdialysis weight gain
KDIGO: Kidney Disease Improving Global Outcomes
LVEF: Left ventricular ejection fraction
MACCE: Main adverse cardiovascular and cerebrovascular event
PTX: Parathyroidectomy
URR: Urea reduction ratio
